# The C-Terminal Domains of the PB2 Subunit of the Influenza A Virus RNA Polymerase Directly Interact with Cellular GTPase Rab11a

**DOI:** 10.1128/jvi.01979-21

**Published:** 2022-03-09

**Authors:** Hana Veler, Haitian Fan, Jeremy R. Keown, Jane Sharps, Marjorie Fournier, Jonathan M. Grimes, Ervin Fodor

**Affiliations:** a Sir William Dunn School of Pathology, University of Oxford, Oxford, United Kingdom; b Diamond Light Source Ltd., Harwell Science and Innovation Campus, Didcot, United Kingdom; c Division of Structural Biology, University of Oxford, Oxford, United Kingdom; d Advanced Proteomics Facility, Department of Biochemistry, University of Oxford, Oxford, United Kingdom; St. Jude Children’s Research Hospital

**Keywords:** influenza virus, RNA polymerase, PB2 polymerase subunit, Rab11a, cytoplasmic trafficking

## Abstract

Influenza A virus (IAV) contains a segmented RNA genome that is transcribed and replicated by the viral RNA polymerase in the cell nucleus. Replicated RNA segments are assembled with viral polymerase and oligomeric nucleoprotein into viral ribonucleoprotein (vRNP) complexes which are exported from the nucleus and transported across the cytoplasm to be packaged into progeny virions. Host GTPase Rab11a associated with recycling endosomes is believed to contribute to this process by mediating the cytoplasmic transport of vRNPs. However, how vRNPs interact with Rab11a remains poorly understood. In this study, we utilized a combination of biochemical, proteomic, and biophysical approaches to characterize the interaction between the viral polymerase and Rab11a. Using pulldown assays, we showed that vRNPs but not complementary RNPs (cRNPs) from infected cell lysates bind to Rab11a. We also showed that the viral polymerase directly interacts with Rab11a and that the C-terminal two-thirds of the PB2 polymerase subunit (PB2-C) comprising the cap-binding, mid-link, 627, and nuclear localization signal (NLS) domains mediate this interaction. Small-angle X-ray scattering (SAXS) experiments confirmed that PB2-C associates with Rab11a in solution forming a compact folded complex with a 1:1 stoichiometry. Furthermore, we demonstrate that the switch I region of Rab11a, which has been shown to be important for binding Rab11 family-interacting proteins (Rab11-FIPs), is also important for PB2-C binding, suggesting that IAV polymerase and Rab11-FIPs compete for the same binding site. Our findings expand our understanding of the interaction between the IAV polymerase and Rab11a in the cytoplasmic transport of vRNPs.

**IMPORTANCE** The influenza virus RNA genome segments are replicated in the cell nucleus and are assembled into viral ribonucleoprotein (vRNP) complexes with viral RNA polymerase and nucleoprotein (NP). Replicated vRNPs need to be exported from the nucleus and trafficked across the cytoplasm to the cell membrane, where virion assembly takes place. The host GTPase Rab11a plays a role in vRNP trafficking. In this study, we showed that the viral polymerase directly interacts with Rab11a mediating the interaction between vRNPs and Rab11a. We mapped this interaction to the C-terminal domains of the PB2 polymerase subunit and the switch I region of Rab11a. Identifying the exact site of Rab11a binding on the viral polymerase could uncover a novel target site for the development of an influenza antiviral drug.

## INTRODUCTION

Influenza A virus (IAV) is a serious threat to human health, causing contagious respiratory disease, usually in the form of seasonal outbreaks. IAV also manifests in pandemics that result in substantial morbidity and mortality (1). The genome of IAV consists of eight negative-sense viral RNA (vRNA) segments, each of which is assembled in a separate ribonucleoprotein (vRNP) complex. In each vRNP complex, the partially complementary 5′ and 3′ vRNA termini are bound to the viral RNA-dependent RNA polymerase, while the rest of vRNA associates with oligomeric nucleoprotein (NP) (2). The IAV polymerase is a heterotrimeric complex composed of polymerase acidic (PA), polymerase basic 1 (PB1), and PB2 subunits. It consists of a core polymerase domain and several peripheral domains, which are connected to the core domain through flexible linkers (3, 4). The PB1 subunit contributes most to the core domain and contains the polymerase active site with the catalytic residues for RNA synthesis. The PA subunit is composed of the N-terminal endonuclease domain (PA-N) and a large PA C-terminal domain (PA-C), which are connected by a linker wrapping around PB1. In contrast to PA-N, PA-C is rather tightly integrated into the polymerase core. The N-terminal third of PB2 (PB2-N) is also part of the polymerase core, while the C-terminal two-thirds (PB2-C) is highly dynamic and composed of discrete domains. These domains include the mid-domain, cap-binding domain, cap-627 linker domain, 627 domain, and the C-terminal nuclear localization signal (NLS) domain. Overall, the polymerase can be viewed as a complex that is made up of a catalytic core (PB1, PA-C, and PB2-N) and flexible and dynamic peripheral appendices (PA-N and PB2-C), which can adopt different positions relative to the polymerase catalytic core. Different conformations, depending on association with viral and cellular factors, underpin the multifunctional nature of the IAV polymerase that acts as both transcriptase and replicase (3–5).

In the course of IAV infection, upon viral entry and fusion of the viral membrane with the endosomal membrane, the eight vRNPs are trafficked into the nucleus of the host cell, where the viral polymerase both transcribes and replicates the vRNA. During transcription the PB2 cap-binding and PA endonuclease domains of the polymerase cleave short capped RNA from cellular capped RNAs which are used to prime viral transcription in a process called cap snatching. Transcription results in capped and polyadenylated viral mRNAs. During replication, the polymerase first synthesizes a cRNA that is assembled with polymerase and NP into complementary RNPs (cRNPs) which then serve as templates for the synthesis of new vRNPs (3, 4). Newly synthesized vRNPs are then exported from the nucleus and trafficked to the plasma membrane for packaging into budding virions (6).

For IAV to be fully infectious, each of the eight vRNPs must be packed in virions. It has been demonstrated that vRNPs establish intersegment interactions to form a supramolecular genomic complex with a 7 + 1 arrangement inside virions (7, 8). While several studies have indicated that specific vRNA-vRNA interactions are likely to contribute to the packaging of the eight vRNPs (9–11), the underlying mechanisms remain to be established. Currently accepted models postulate that genome assembly is intimately linked to vRNP transport across the cytoplasm, a process that involves the cellular protein Rab11a (6, 12, 13). Rab11a belongs to a family of small GTPases involved in apical trafficking of recycling endosomes which sort and transport cargo destined for release from the apical cell membrane (14). Rab11a recruits molecular motor proteins to the recycling endosome through interactions with its effectors, the Rab11 family-interacting proteins (Rab11-FIPs) (15). Rab11-FIPs can associate with both actin and microtubule-associated motor proteins, indicating that Rab11a-associated recycling endosomes can use multiple cytoskeletal networks for transport. In cells infected with IAV, Rab11a was shown to colocalize with vRNPs and knockdown of Rab11a or overexpression of a dominant negative Rab11a mutant decreased viral replication, leading to the suggestion that vRNPs are transported to the plasma membrane along cytoskeletal filaments via an interaction with Rab11a in recycling endosomes (16–21). More recently, an alternative model has been proposed in which Rab11a and vRNPs are recruited to membranes of remodeled endoplasmic reticulum (ER), leading to the biogenesis of irregularly coated vesicles (ICVs), which ensure transport of vRNPs from the ER to the plasma membrane (22). Rab11a has also been implicated in the formation of liquid organelles promoting genome assembly and mediating cell-cell spread and reassortment of IAV genomes via tunneling nanotubes (23–25).

In spite of Rab11a playing a key role in vRNP trafficking and potentially also in facilitating genome assembly and reassortment of IAV genomes, little is known about the specific interactions mediating vRNP-Rab11a associations. IAV vRNPs were shown to associate with Rab11a in pulldown assays using infected cell lysates, while pulldown assays using lysates from cells expressing individual vRNP components suggested that the PB2 polymerase subunit but no other protein component of vRNP alone interacted with Rab11a (18). This result highlighted the viral polymerase, and the PB2 subunit in particular, as a mediator of the interaction between vRNP and Rab11a. This idea received further support from studies using fluorescence resonance energy transfer microscopy, which indicated a distance of <10 nm between PB2 and Rab11 in IAV-infected cells, suggesting that they might directly interact (26).

In this study, we utilized a combination of biochemical, proteomic, and structural approaches to characterize the interaction between the IAV polymerase and Rab11a. We show that Rab11a specifically interacts with vRNPs but not cRNPs from infected cell lysates. We also show that the interaction between the viral polymerase and Rab11a is direct and that the 627 domain of PB2 is required for this interaction. While the isolated 627-NLS domain fails to bind to Rab11a, PB2-C binds directly to Rab11a. Our observations provide novel insights into the interaction of influenza virus vRNPs with cellular GTPase Rab11a and expand our current view of the role of PB2 in this interaction.

## RESULTS

### Recombinant Rab11a interacts with vRNPs.

**FIG 1:**
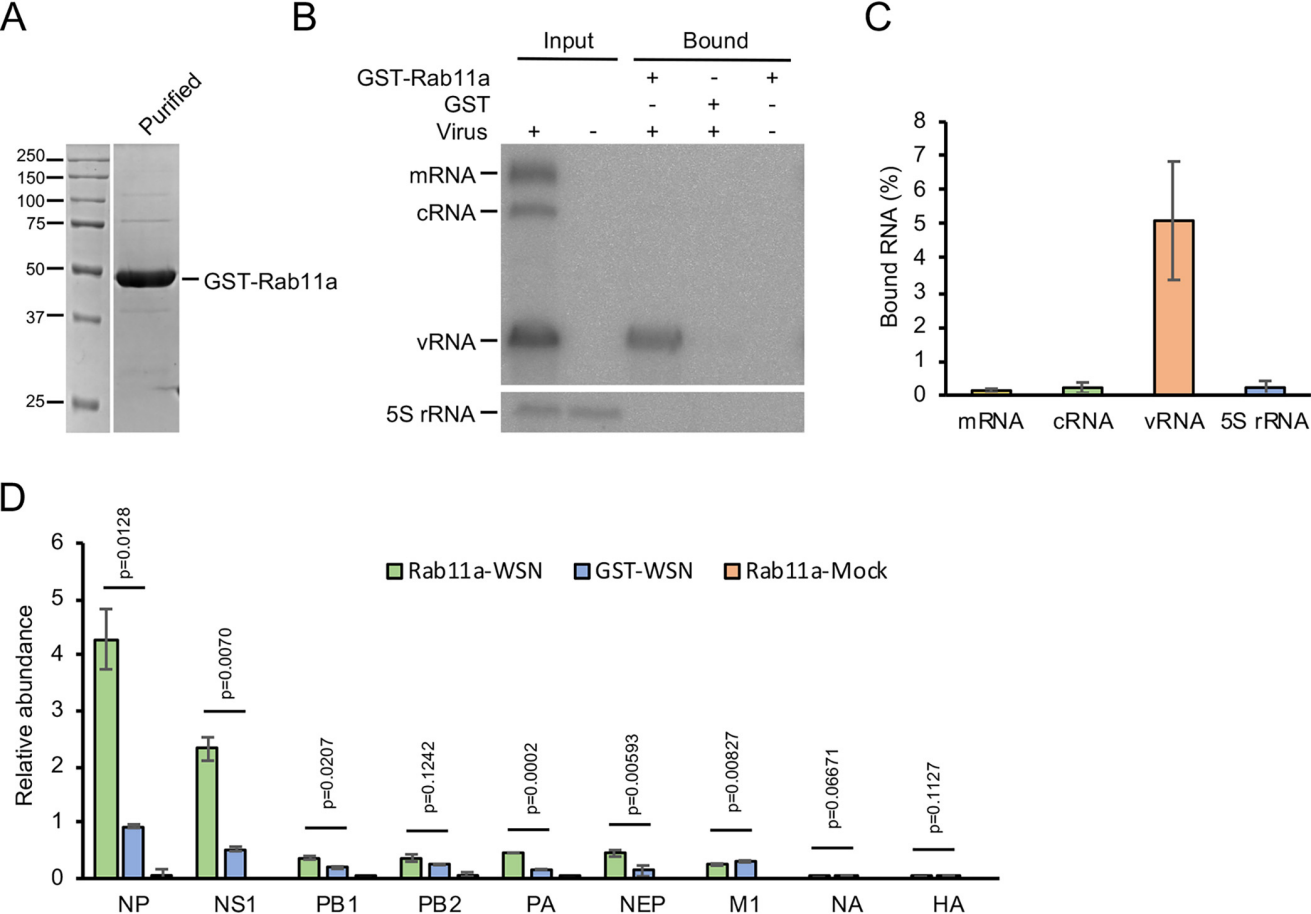
Recombinant Rab11a binds vRNPs from infected cell lysates. (A) Recombinant GST-Rab11a was analyzed by SDS-PAGE and proteins were visualized by staining with Coomassie brilliant blue. (B) HEK 293T cells were infected with influenza A/WSN/1933 (H1N1) (WSN) virus at a multiplicity of infection (MOI) of 5 or were mock infected, and whole-cell lysates were isolated at 12 h postinfection. Lysates were incubated with immobilized GST-Rab11a or GST. Viral RNAs isolated from cell lysates (input) and material bound to GST-Rab11a (bound) were analyzed by primer extension. 5S rRNA was used as a control. Note that 10-fold less cell lysate was used to prepare the input sample than for bound samples. (C) Quantitative analysis of viral RNAs bound to GST-Rab11a or GST. Data are means ± standard deviations (*n* = 3 independent experiments). (D) Mass spectrometry-based proteomic analysis and label-free relative quantification of viral proteins bound to Rab11a or GST. A two-tailed *t* test was used to calculate *P* values (*n* = 2 independent experiments).

To analyze the interaction of vRNP with Rab11a, we first addressed whether we could recapitulate the interaction using purified recombinant Rab11a *in vitro*. We expressed human Rab11a as a fusion protein with a glutathione *S*-transferase (GST) affinity tag at its N terminus separated by a human rhinovirus (HRV) 3C protease (3C) cleavage site. Since the interaction between vRNPs and Rab11a has been reported to require an active, GTP-bound conformation of Rab11a (18, 20, 21), we used Rab11a with a Q70L substitution to favor the GTP-bound form (27). GST-Rab11a was purified as a fusion protein by affinity and size exclusion chromatography (SEC), and the purified protein was analyzed by SDS-PAGE (Fig. 1A). We then used a GST pulldown assay in which GST-Rab11a was immobilized on glutathione Sepharose beads to test its interaction with vRNPs. HEK-293T cells were mock infected or infected with influenza A/WSN/1933 (H1N1) virus (here referred to as WSN) and lysed at 12 h postinfection (hpi), and the lysates were subjected to a GST pulldown assay. First, we asked whether vRNA was bound to Rab11a by analyzing neuraminidase (NA)-specific RNAs by primer extension (Fig. 1B). The 5S rRNA was monitored as a control. NA mRNA, cRNA, and vRNA were detected in the infected cell lysates but not in the uninfected lysate (Fig. 1B). We detected vRNA in the GST-Rab11a-bound fraction of WSN-infected lysates; however, only background levels of cRNA, mRNA, and 5S rRNA were detected (Fig. 1B). On average, about 5% of total vRNA from cell lysates was recovered in the GST pulldown assay, while less than 0.3% of cRNA, mRNA, and 5S rRNA were present (Fig. 1C). We further used a mass spectrometry-based shotgun proteomic approach to identify proteins bound to recombinant purified GST-Rab11a after incubation with WSN-infected cell lysates. We found that all four protein components of vRNPs, i.e., PB1, PB2, PA, and NP, were enriched in samples bound to GST-Rab11a compared to the GST control (Fig. 1D). Interestingly, viral nonstructural protein 1 (NS1) and nuclear export protein (NEP) (also known as nonstructural protein 2 [NS2]) were also enriched. While other viral proteins such as hemagglutinin (HA), NA, and matrix protein 1 (M1) were also detected, these proteins were not enriched in the GST-Rab11a sample compared to the GST tag alone, suggesting that their interaction is nonspecific. Taken together, these data show that Rab11a specifically interacts with influenza virus vRNPs.

### Influenza A virus polymerase binds directly to Rab11a.

**FIG 2:**
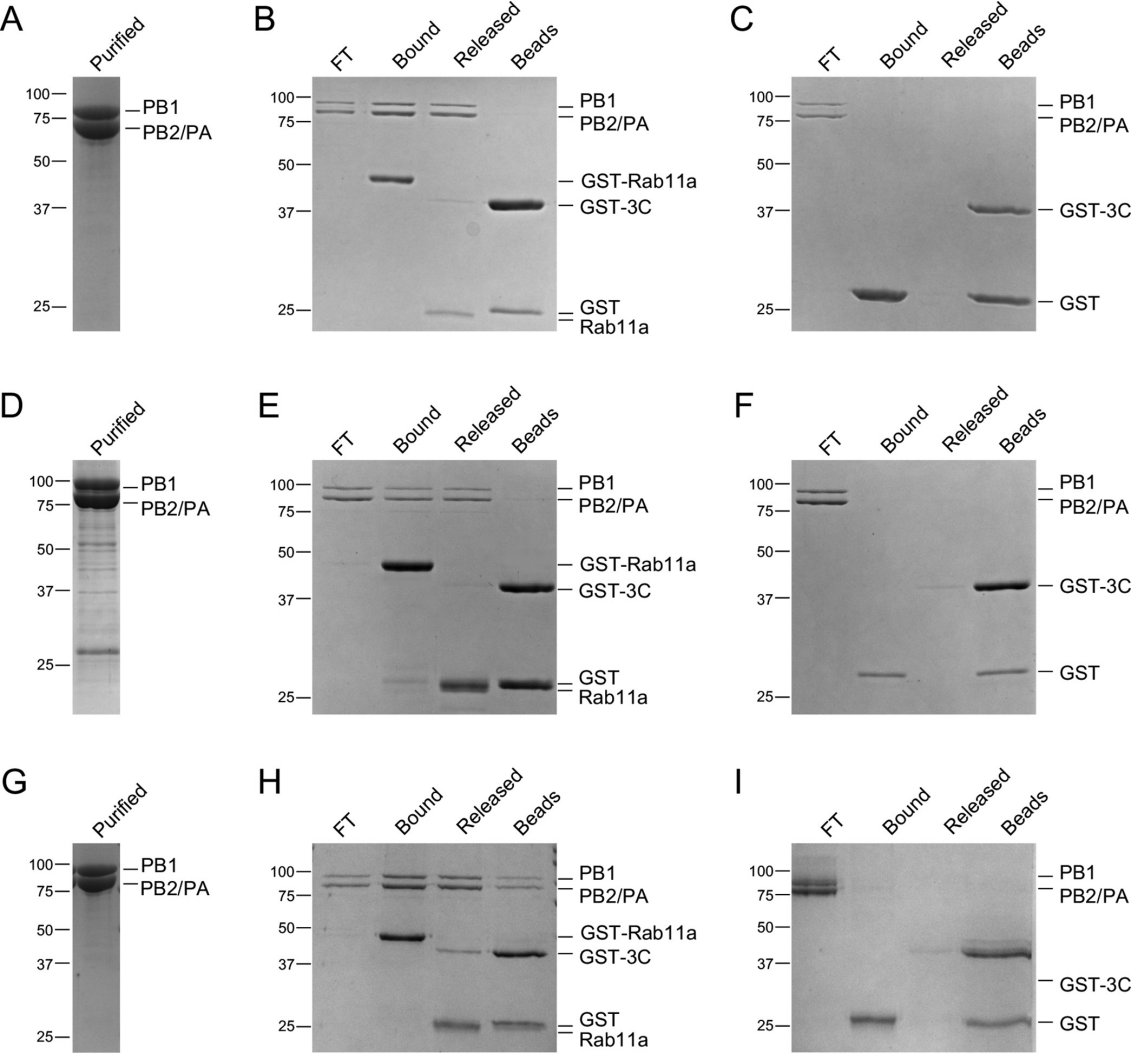
The RNA polymerase of IAV directly interacts with Rab11a. Purified recombinant polymerase from A/Northern Territory/60/1968 (H3N2) (NT60) (A), A/Brevig Mission/1/1918 (H1N1) (BM18) (D), and A/duck/Fujian/01/2002 (H5N1) (Fj02) (G). GST-Rab11a (B, E, and H), and GST tag (C, F, and I) were immobilized on glutathione Sepharose beads before being incubated with a purified NT60 (B and C), BM18 (E and F) or Fj02 (H and I) polymerase. Proteins were released using GST-tagged 3C protease (GST-3C) to cleave off Rab11a from the GST tag. Purified polymerase (purified), unbound flowthrough polymerase (FT), proteins bound to GST-Rab11a or GST tag (bound), proteins released upon cleavage with GST-tagged 3C protease (GST-3C) (cleaved), and proteins remaining associated with the beads after cleavage (beads) were analyzed by SDS-PAGE, and proteins were visualized by staining with Coomassie brilliant blue.

Having found that vRNPs specifically associate with recombinant Rab11a, we next used the same pulldown assay to address whether the viral polymerase mediates the interaction between Rab11a and vRNP. We coexpressed the three subunits of the influenza A/Northern Territory/60/1968 (H3N2) virus polymerase (here referred to as NT60 polymerase) in insect cells using a recombinant baculovirus and purified the NT60 polymerase as described previously (28, 29) (Fig. 2A). The interaction between GST-Rab11a and NT60 polymerase was then tested in a GST pulldown assay. SDS-PAGE analysis of pulldown fractions showed that NT60 polymerase bound to immobilized GST-Rab11a and was released from the beads together with Rab11a upon cleavage with 3C protease (Fig. 2B). As a control, we incubated purified NT60 polymerase with immobilized GST tag, which showed no polymerase binding (Fig. 2C).

To further assess the association between polymerase and Rab11a, we purified recombinant heterotrimeric polymerase from different IAV subtypes, i.e., the pandemic influenza A/Brevig Mission/1/1918 (H1N1) virus (here referred to as the BM18 polymerase) (Fig. 2D) and an avian influenza A/duck/Fujian/01/2002 (H5N1) virus (here referred to as Fj02 polymerase) (Fig. 2G). Both the BM18 and Fj02 polymerases bound to immobilized GST-Rab11a (Fig. 2E and H). There was no binding of the BM18 and Fj02 polymerase to the immobilized GST tag (Fig. 2F and I).

Together, these data show that IAV polymerase and Rab11a can form a complex *in vitro*, which is consistent with previous reports showing that vRNPs interact with Rab11a through the polymerase and suggestions that this interaction is direct (18, 21, 26). Moreover, these data also establish that the IAV polymerase-Rab11a interaction is direct and independent of IAV subtype.

### The C-terminal domains of PB2 (PB2-C) mediate the interaction of the influenza A virus polymerase with Rab11a.

**FIG 3:**
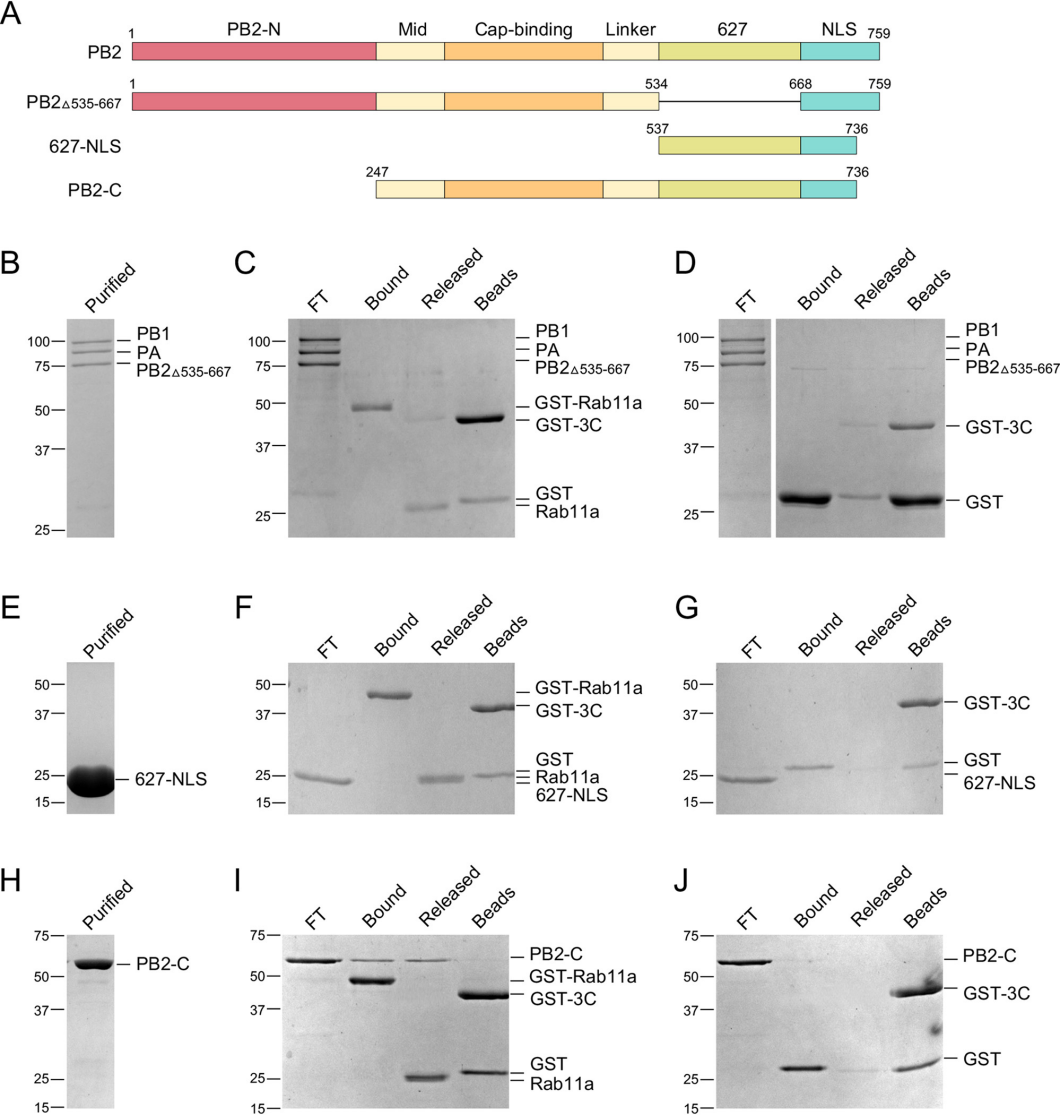
A complete C-terminal region of the PB2 polymerase subunit is required for Rab11a binding. (A) Schematics of full-length PB2, PB2 lacking the 627 domain (PB2_Δ535–667_), and PB2 fragments 627-NLS and PB2-C. (B to D) The 627 domain of PB2 is required for interaction between the influenza virus polymerase and Rab11a. (E to G) The PB2 627-NLS domain alone does not bind Rab11a. (H to J) The C-terminal domains of PB2 (PB2-C) mediate interaction with Rab11a. (B, E, and H) Purified recombinant polymerase from A/Northern Territory/60/1968 (H3N2) (NT60) lacking the 627 domain (PB2_Δ535–667_) (B) and purified recombinant 627-NLS (E) and PB2-C (H) fragments of NT60 PB2. GST-Rab11a (C, F, and I) or GST tag (D, G, and J) were immobilized onto glutathione Sepharose beads before being incubated with a purified PB2_Δ535–667_ (C and D), 627-NLS (F and G), or PB2-C (I and J). Proteins were released by using GST-tagged 3C protease (GST-3C) to cleave off Rab11a from the GST tag. Purified polymerase or polymerase fragments (purified), unbound flowthrough polymerase or fragments (FT), proteins bound to GST-Rab11a or GST tag (bound), proteins released upon cleavage with GST-tagged 3C protease (GST-3C) (cleaved), and proteins remaining associated with the beads after cleavage (beads) were analyzed by SDS-PAGE, and proteins were visualized by staining with Coomassie brilliant blue.

Having shown that the IAV polymerase directly interacts with Rab11a, we then addressed whether this interaction occurs through the PB2 subunit of the polymerase as has been proposed (18, 26). Previously, a recombinant influenza A virus polymerase lacking the PB2 627 domain, which has no detectable activity in a cell-based minireplicon assay, was shown to form a heterotrimeric complex and exhibit polymerase activity *in vitro* (30). Therefore, we designed a baculovirus expressing NT60 polymerase lacking the PB2 627 domain (PB2_Δ535–667_) (Fig. 3A). The NT60 PB2_Δ535–667_ polymerase was expressed in insect cells and purified as described previously (28, 29) (Fig. 3B). Interestingly, the polymerase lacking the PB2 627 domain failed to form a complex with Rab11a (Fig. 3C). No NT60 PB2_Δ535–667_ polymerase bound to immobilized GST tag either (Fig. 3D). These results demonstrate that the PB2 627 domain is required for the interaction between the influenza virus polymerase and Rab11a.

To address whether the PB2 627 domain is sufficient for mediating polymerase interaction with Rab11, we generated a PB2 construct from the influenza virus NT60 polymerase expressing amino acid residues 537 to 736, which correspond to the PB2 627 and NLS domains (here referred to as the NT60 627-NLS) but lack the flexible NLS peptide (residues 737 to 759) (Fig. 3A). NT60 627-NLS was expressed in Escherichia coli as a fusion protein with an N-terminal GST tag separated by a 3C cleavage site. Purified NT60 627-NLS was analyzed by SDS-PAGE (Fig. 3E). SDS-PAGE analysis of pulldown fractions showed that the NT60 627-NLS domain does not form a complex with immobilized GST-Rab11a (Fig. 3F). There was also no binding between NT60 627-NLS and immobilized GST tag (Fig. 3G). Together, these results show that while the PB2 627 domain is required for the interaction between the IAV polymerase and Rab11a, the isolated 627-NLS domain alone is not sufficient for the interaction with Rab11a. Based on these findings, we hypothesized that other PB2 domains are required to form a complex with Rab11a.

To test this hypothesis, we generated a PB2 construct from the influenza virus NT60 polymerase expressing amino acid residues 247 to 736, which correspond to the C-terminal two-thirds of PB2 (PB2-C) but lack the flexible C-terminal NLS peptide (residues 737 to 759) (Fig. 3A). NT60 PB2-C was expressed and purified like 627-NLS. Purified NT60 PB2-C was analyzed by SDS-PAGE (Fig. 3H). SDS-PAGE analysis of pulldown fractions showed that NT60 PB2-C bound to immobilized GST-Rab11a and was released together with Rab11a upon 3C cleavage from the beads (Fig. 3I). There was no binding between NT60 PB2-C and immobilized GST tag (Fig. 3J).

Overall, these results show that PB2-C binds directly to Rab11a, suggesting that PB2-C is the major determinant of the interaction between the IAV polymerase and Rab11a. These results are consistent with previous proposals that the interaction between the IAV polymerase and Rab11a occurs through the PB2 subunit (18, 26).

### The switch I region of Rab11a is important for PB2 binding.

**FIG 4:**
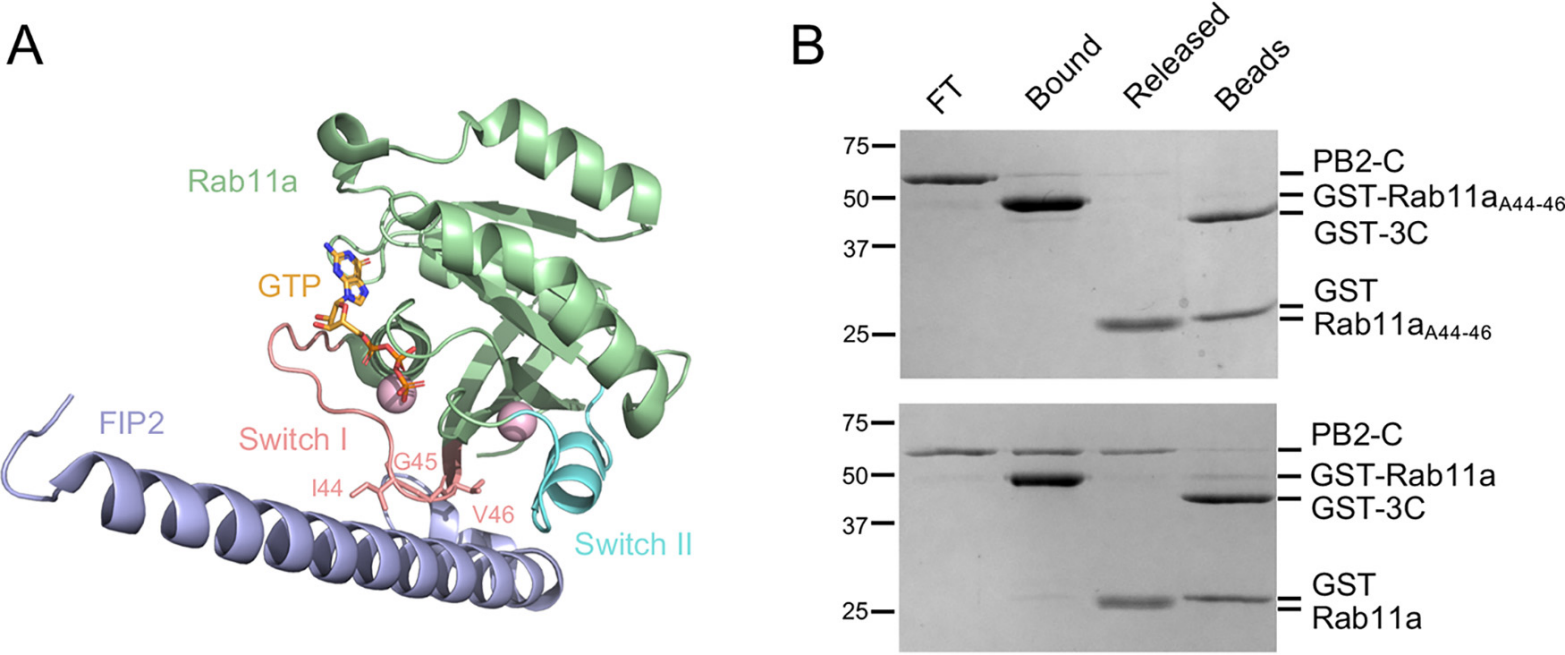
The switch I region of Rab11a is important for PB2 binding. (A) Crystal structure of Rab11a in complex with Rab11 family-interacting protein 2 (Rab11-FIP2) showing the involvement of the switch I region in the interaction. The three amino acids in the switch I region at the Rab11-FIP2 interaction interface are indicated. Mg^2+^ ions are shown as pink spheres. The PDB code is 2GZD. (B) GST-Rab11a_A44-46_ mutant (top) and wild-type Rab11a (bottom) were immobilized on glutathione Sepharose beads and incubated with PB2-C derived from the polymerase of influenza A/Northern Territory/60/1968 (H3N2) (NT60) virus. Proteins were released by using GST-tagged 3C protease (GST-3C) to cleave off Rab11a from the GST tag. Unbound flowthrough PB2-C (FT), proteins bound to GST-Rab11a_A44-46_ or Rab11a (bound), proteins released upon cleavage with GST-tagged 3C protease (GST-3C) (cleaved), and proteins remaining associated with the beads after cleavage (beads) were analyzed by SDS-PAGE, and proteins were visualized by staining with Coomassie brilliant blue.

The crystal structure of Rab11-FIP2 in complex with Rab11a shows that the binding interface involves the switch I and switch II regions of Rab11a (31) (Fig. 4A). To test whether PB2-C interacts with the same region of Rab11a as Rab11-FIP2, we mutated amino acid residues 44 to 46 to alanines (IGV to AAA) in the switch I region. The Rab11a switch I mutant (here referred to as Rab11a_A44–46_) was expressed and purified as a fusion protein with an N-terminal GST tag. In a pulldown assay with immobilized GST-Rab11a_A44–46_, only trace amounts of PB2-C were found to bind (Fig. 4B, top). In contrast, PB2-C bound to immobilized wild-type GST-Rab11a as expected (Fig. 4B, bottom). Hence, mutations in the switch I of Rab11a reduce the interaction of Rab11a with PB2-C. These data suggest that PB2 binds to Rab11a at a site at which Rab11-FIP2 also binds. Moreover, these data are consistent with findings that influenza virus infection modulates host recycling by competing with Rab11-FIPs for interaction with Rab11a (32).

### PB2-C forms a compact folded protein complex with Rab11a in solution.

**FIG 5:**
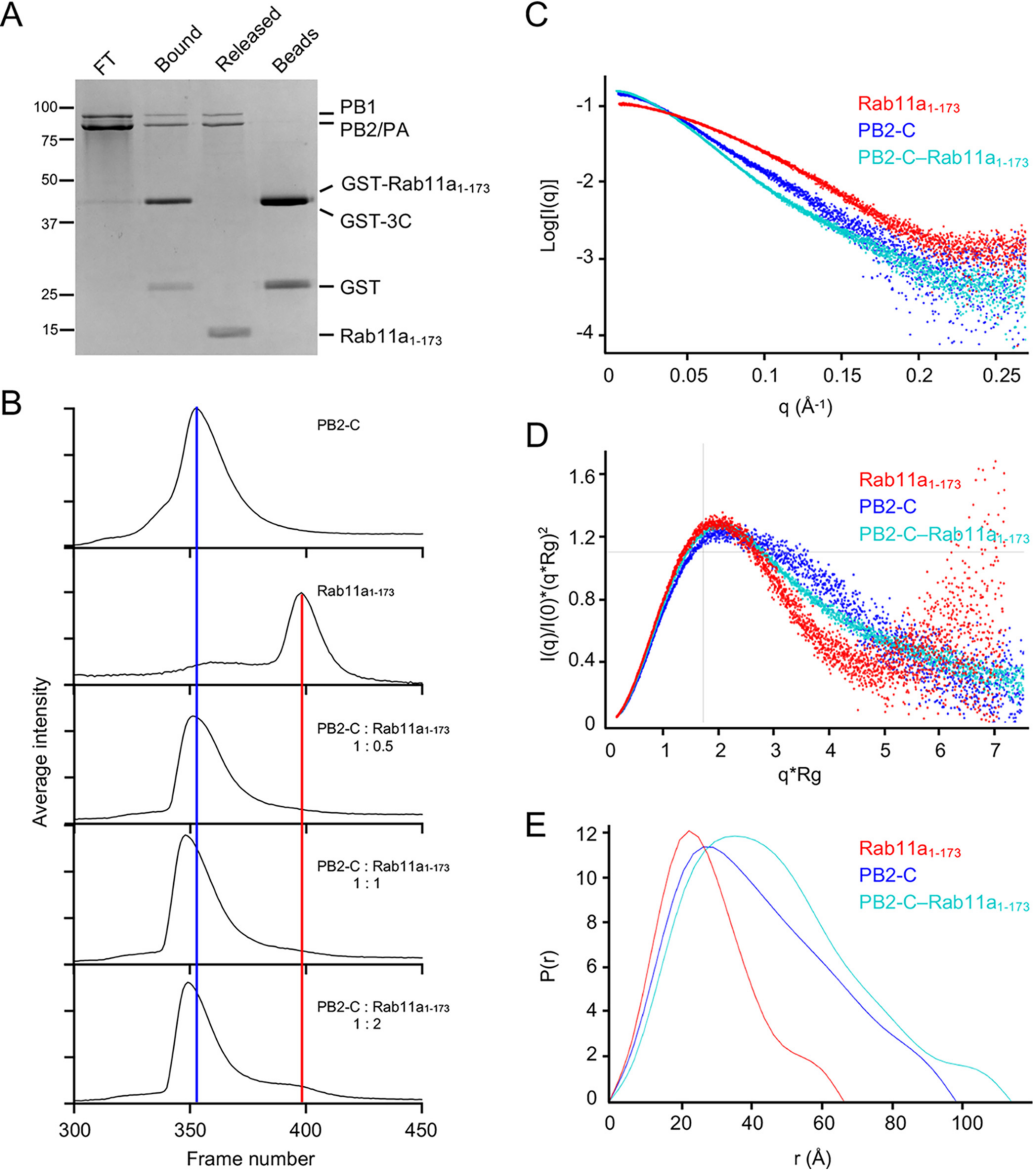
PB2-C and Rab11a_1-173_ form a 1:1 stoichiometric complex in solution. (A) The C-terminal disordered tail of Rab11a is not required for IAV polymerase binding. Recombinant truncated GST- Rab11a_1-173_ lacking the C-terminal 43 amino acid residues was immobilized on glutathione Sepharose beads and incubated with polymerase of influenza A/Northern Territory/60/1968 (H3N2) (NT60) virus. Proteins were released by using GST-tagged 3C protease (GST-3C) to cleave off Rab11a from the GST tag. Unbound flowthrough polymerase (FT), proteins bound to GST-Rab11a_1-173_ (bound), proteins released upon cleavage with GST-tagged 3C protease (GST-3C) (cleaved), and proteins remaining associated with the beads after cleavage (beads) were analyzed by SDS-PAGE, and proteins were visualized by staining with Coomassie brilliant blue. (B to E) SEC-SAXS analysis of Rab11a, PB2-C, and PB2-C–Rab11a_1-173_ complex. Chromatograms from the differential refractive index detector are shown for PB2-C, Rab11a, and the PB2-C–Rab11a_1-173_ complex at 1:0.5, 1:1, and 1:2 molar ratios. Blue and red vertical lines indicate PB2-C and Rab11a_1-173_ elution peaks, respectively (B). Scattering profiles, Kratky plots, and pair distance distribution function of Rab11a_1-173_, PB2-C, and the PB2-C–Rab11a_1-173_ complex are normalized and shown in panels C to E, respectively.

To validate our pulldown assay results, we used small-angle X-ray scattering (SAXS). Since the C-terminal 43 amino acid residues of Rab11a are disordered, we generated a truncated Rab11a lacking the C-terminal 43 residues (Rab11a_1-173_), the lack of which has been shown previously to have no impact on protein folding (31, 33). To confirm that truncation of the C-terminal 43 amino acid residues of Rab11a does not disturb the interaction with the IAV virus polymerase, we incubated GST-Rab11a_1-173_ with purified NT60 polymerase. As expected, truncated Rab11a_1-173_ formed a complex with the NT60 polymerase (Fig. 5A), demonstrating that the C-terminal 43 amino acid residues of Rab11a are not required for the interaction between the IAV polymerase and Rab11a.

Next we examined the interaction between truncated Rab11a_1-173_ and PB2-C from influenza A/Vietnam/1203/2004 (H5N1) virus polymerase (Vn04 PB2-C) by SEC coupled to SAXS (SEC-SAXS). To determine the point of saturation of PB2-C with Rab11a, SEC-SAXS data of PB2-C–Rab11a complex were collected across a range of molar ratios of Rab11a to PB2-C (0.5:1, 1:1, and 2:1 [Fig. 5B]). There was a shift in the position of the PB2-C peak with a 0.5-fold molar excess of Rab11a_1-173_, and no peak was observed at the expected position of Rab11a_1-173_ alone, confirming that all Rab11a_1-173_ was eluted as a complex with PB2-C. When an equimolar amount of Rab11a_1-173_ was added to PB2-C, there was a further shift in peak position, suggesting a concentration dependence in elution position. At a 2-fold molar excess of Rab11a_1-173_, PB2-C was saturated, as no further shift in the position of the PB2-C–Rab11a_1-173_ peak was observed, and a peak corresponding to Rab11a_1-173_ appeared. These results suggest the formation of a PB2-C–Rab11a_1-173_ complex at a 1:1 molar ratio.

The SEC-SAXS data at the peak area were buffer subtracted and analyzed in ScÅtter (http://www.bioisis.net). Analysis of the individual Rab11a_1-173_ and PB2-C scattering data showed that the estimated molecular weight is in agreement with each protein being monomeric, and a Kratky plot shows that each protein is compactly folded in solution (Table 1 and Fig. 5C and D). Estimation of the molecular weight for the complex peak gives a molecular weight of 84.6 kDa, in good agreement with the predicted molecular weight of a 1:1 complex of 78.6 kDa (Table 1). Analysis of the scattering data using a Kratky plot suggested that the complex is well folded in solution and even appears more compact than the PB2-C alone (Fig. 5D). From the radius of gyration (R_g_), maximum dimension of particle (D_max_), and the paired-distance distribution plot, it appears that Rab11a_1-173_ binds near the center of PB2-C, as the increase to the Dmax and Rg is much less than would be expected if it was binding to an end and dramatically increasing the length of the PB2-C (Table 1 and Fig. 5E). Though the high-resolution structures of the two components are known, the relative orientations of these could not be unambiguously modeled from the SAXS data.

**TABLE 1:** SAXS analysis of Rab11a_1-173_, PB2-C, and PB2-C–Rab11a_1-173_ complex

Sample	R_g_^*a*^ (Å)	D_max_^*b*^ (Å)	MW^*c*^ (kDa)	MW, theoretical^*d*^ (kDa)
Rab11a_1-173_	22.8	66.0	24.4	19.4
PB2-C	32.9	97.5	58.2	59.2
Complex	36.9	113.0	84.6	78.6

a R_g_, radius of gyration estimated from Guinier analysis.

b D_max_, maximum dimension of particle estimated from paired-distance distribution [P(r)] function.

c MW, molecular weight, estimated from Porod volume determination.

d Theoretical MW for each component or the complex in a 1:1 manner.

## DISCUSSION

In this study, we investigated the interaction between the IAV RNA polymerase and cellular GTPase Rab11a. We show that vRNPs associate with Rab11a and provide biochemical evidence that the IAV polymerase mediates this association by directly interacting with Rab11a. Using recombinant trimeric IAV polymerases from various IAV subtypes, we demonstrated that Rab11 binding is a general property of IAV polymerases. A polymerase lacking the PB2 627 domain failed to bind to Rab11a, indicating that the PB2 627 domain is required for the interaction. However, an isolated 627-NLS domain was not sufficient for the interaction. In contrast, PB2-C, corresponding to the C-terminal two-thirds of PB2, encompassing the cap-binding, mid-link, 627, and NLS domains, bound Rab11a. Small-angle X-ray scattering data confirmed that PB2-C forms a stable heterodimeric complex with Rab11a in solution, with a compact well-folded structure. Thus, our study links Rab11a binding and, consequently, genome trafficking and assembly to a particular region of the IAV polymerase, the flexible C-terminal region of PB2. The 627 domain of PB2-C has recently been shown to be directly involved in mediating interactions with a central part of the host protein ANP32A (34), a factor that plays a key role in the replication of the IAV genome in the host cell nucleus. Therefore, PB2-C appears to play a major role in mediating polymerase interactions with different host factors during different stages of the virus life cycle in different cellular compartments. Our data demonstrating a direct interaction between the C-terminal region of PB2 and Rab11a are in full agreement with previous studies suggesting that the interaction between the polymerase and Rab11a likely occurs via the PB2 subunit (18, 26).

In an attempt to delineate the region of Rab11a involved in IAV polymerase binding, we investigated the role of the switch I region, previously shown to be involved in binding Rab11-FIP2 and Rab11-FIP3 (31, 33). Interestingly, we found that the same region that was shown to be important for binding Rab11-FIPs, amino acids 44 to 46 in the switch I region, are also important for IAV polymerase binding. Given that Rab11-FIP2 and Rab11-FIP3 bind to the same amino acid residues of Rab11a as PB2-C, our results provide further support for the idea that binding of IAV vRNP segments potentially outcompetes Rab11-FIPs for binding to Rab11a, leading to altered transport of Rab11a in IAV-infected cells. Altered Rab11a transport, in turn, could lead to clustering of single- and double-membrane vesicles and generation of hot spots for vRNP interactions and genome assembly as proposed previously (32, 35).

In line with previous observations that vRNA colocalizes and immunoprecipitates with Rab11a (18), we found that vRNPs bind to Rab11a. However, intriguingly, cRNPs were not bound even though they were present in the lysates. The question arises how this specificity is achieved considering that vRNPs and cRNPs are composed of identical viral proteins. It is unlikely that Rab11a would directly bind vRNA but not cRNA. Rather, we speculate that this differential binding could be the result of the sequence differences in the vRNA and cRNA termini that are bound by the polymerase in vRNPs and cRNPs. In fact, it has been shown that vRNA and cRNA can occupy different binding sites on the surface of the polymerase; while the 3′ terminus of vRNA can bind at two different sites, the 3′ terminus of cRNA appears to be able to bind only one of these (29, 36–38). Differences in RNA binding might, in turn, promote polymerase association with different sets of viral and host factors. This could also explain why only vRNPs and not cRNPs appear to be exported from the nucleus; binding of viral NEP and M1, which mediate interaction of vRNPs with the cellular export factor CrmI, might not be compatible with cRNA-bound polymerase (6, 39). We observed NEP associating with Rab11a, suggesting that NEP might remain associated with vRNPs after nuclear export, throughout cytoplasmic trafficking. Intriguingly, the viral NS1 protein was also observed to specifically bind Rab11a, suggesting that it might also be present in vRNP complexes destined for packaging into virus particles. In line with this, NS1 was reported to bind to NP in vRNPs and enhance genome packaging (40, 41). The association of NEP and NS1 with vRNPs during cytoplasmic trafficking could also offer an explanation for the incorporation of these viral proteins, formerly considered nonstructural, into virions (41, 42).

Taken together, the results of this study show that the IAV RNA polymerase interacts directly with the cellular GTPase Rab11a and that the highly mobile C-terminal domains of the PB2 polymerase subunit mediate the interaction. Additionally, our data also show that vRNPs but not cRNPs interact with Rab11a and that the viral NEP and NS1 proteins might be present in vRNP-Rab11a complexes. These findings expand our understanding of the cytoplasmic transport of the influenza virus vRNPs as well as identify the molecular details of Rab11a binding on the viral polymerase, which suggest a novel target site for the development of an influenza antiviral drug.

## MATERIALS AND METHODS

### Cells.

Human embryonic kidney 293T (HEK-293T), Madin-Darby canine kidney (MDCK), and Spodoptera frugiperda 9 (Sf9) insect cells were sourced from the Cell Bank of the Sir William Dunn School of Pathology, University of Oxford, and the Division of Structural Biology, University of Oxford. Cell culture media were purchased from Sigma-Aldrich unless otherwise specified. HEK-293T and MDCK cells were grown in Dulbecco’s modified Eagle medium (DMEM) supplemented with 10% (vol/vol) fetal calf serum (FCS). Cells were maintained at 37°C and 5% CO_2_. Sf9 cells were grown in Sf-900 II serum free medium (Gibco) supplemented with penicillin-streptomycin (Gibco). Cell density was maintained between 0.5 million and 2 million cells mL^−1^. Cells were maintained at 27°C, with shaking at 110 rpm. Cell lines have not been authenticated but tested negative for mycoplasma contamination.

### Viruses.

Recombinant influenza A/WSN/33 (H1N1) (WSN) virus was generated using the eight-plasmid system (43). Approximately 10^6^ HEK-293T cells were transfected with 1 μg each of pHW2000-PB2, pHW2000-PB1, pHW2000-PA, pHW2000-HA, pHW2000-NP, pHW2000-NA, pHW2000-M, and pHW2000-NS using Lipofectamine 2000 transfection reagent (Thermo Fisher Scientific) and Opti-MEM according to the manufacturer’s instructions. At 24 h posttransfection, DMEM containing 10% (vol/vol) FCS was replaced with DMEM containing 0.5% (vol/vol) FCS. Cell culture medium containing virus was harvested 48 h after changing of the medium. The virus titer was determined by plaque assay in MDCK cells.

### Protein expression and purification.

Subunits of the influenza A/Northern Territory/60/1968 (H3N2) (NT60), A/Brevig Mission/1/1918 (H1N1) (BM18), and A/duck/Fujian/01/2002 (H5N1) (Fj02) virus polymerases were expressed in Sf9 cells from codon-optimized genes (GeneArt or Synbio) using the MultiBac system (44). Expression and purification of protein complexes were performed as described previously (28, 29, 45). The construct to express the polymerase of the NT60 virus with a deleted PB2 627 domain (amino acid residues 535 to 667) was generated by PCR site-directed mutagenesis. The Rab11a gene (UniProtKB: P62491) was codon optimized for expression in Spodoptera frugiperda insect cells (GeneArt) and cloned into the pGEX-6P-1 expression vector with an N-terminal GST tag separated by an HRV 3C protease cleavage site. Constructs to express constitutively active Rab11a with a Q70L mutation (Rab11CA), a switch I mutant (I44A, G45A, and V46A) (Rab11a_A44–46_), and C-terminally truncated Rab11a including amino acids 1 to 173 (Rab11a_1-173_) were generated by PCR mutagenesis. Constructs to express NT60 PB2 fragments 627-NLS (amino acids 537 to 736) and PB2-C (amino acids 247 to 736) lacking the flexible NLS peptide (amino acids 737 to 759) were generated by PCR and cloned into the pGEX-6P-1 expression vector with an N-terminal GST tag and 3C cleavage site. GST-Rab11a, GST-Rab11a_A44–46_, and GST-Rab11a_1-173_ proteins were expressed in E. coli BL21(DE3) cells in lysogeny broth (LB) medium. Cells were cultured at 37°C to an optical density at 600 nm (OD_600_) of 0.6 and induced with isopropyl β-d-1-thiogalactopyranoside (IPTG) to a final concentration of 1 mM at 37°C for 6 h. 627-NLS and PB2-C proteins were expressed in E. coli Rosetta (DE3) cells in 2× yeast extract tryptone (YT) medium. Cells were cultured at 37°C to an OD_600_ of 0.6 and induced with IPTG to a final concentration of 0.5 mM at 18°C for 16 h. Cells were harvested by centrifugation at 4,000 × *g* for 20 min and lysed by sonication in lysis buffer (50 mM HEPES-NaOH [pH 7.5], 500 mM NaCl, 10% [vol/vol] glycerol, 0.05% [wt/vol] octyl β-d-1-thioglucopyranoside [OTG], 1 mM dithiothreitol [DTT], 1 mg mL^−1^ lysozyme). Cell debris was removed by centrifugation at 35,000 × *g* for 45 min at 4°C. GST-tagged proteins were purified using glutathione Sepharose 4B beads (GE Healthcare). GST-Rab11a and GST-Rab11a_A44–46_ proteins were eluted overnight at 4°C in elution buffer (50 mM HEPES-NaOH [pH 7.5], 500 mM NaCl, 10% [vol/vol] glycerol, 0.05% [wt/vol] OTG, 25 mM reduced glutathione). Rab11a_1-173_, 627-NLS, and PB2-C proteins were released from the beads overnight at 4°C in wash buffer (50 mM HEPES-NaOH [pH 7.5], 500 mM NaCl, 10% [vol/vol] glycerol, 0.05% [wt/vol] OTG) supplemented with 1 mM DTT and 0.5 mg 3C protease. The supernatants containing proteins were collected and concentrated using an Amicon Ultra centrifugal filter unit (Merck Millipore; nominal molecular weight limit [NMWL] of 30 kDa). The concentrated proteins were applied onto a Superdex 200 Increase 10/300 GL column (GE Healthcare) connected to an Äkta chromatography system (GE Healthcare) at 4°C equilibrated with a buffer consisting of 25 mM HEPES-NaOH (pH 7.5), 500 mM NaCl, and 5% (vol/vol) glycerol. The fractions containing proteins were pooled and concentrated as done previously. The molecular weight and the purity of the proteins were determined by SDS-PAGE with Coomassie blue staining. The proteins were flash-frozen using liquid nitrogen and stored at −80°C. To express PB2-C (amino acids 247 to 736) from influenza A/Vietnam/1203/2004 (H5N1) (Vn04) virus (GenBank accession number AY818126.1), the coding region was codon optimized for expression in E. coli cells (GeneArt) and cloned into pET151/d-TOPO bacterial vector. Vn04 PB2-C was expressed in E. coli Rosetta (DE3) cells in 2× YT medium. Cells were harvested by centrifugation at 4,000 × *g* for 20 min and lysed by sonication in lysis buffer (50 mM Tris-HCl [pH 8.0], 200 mM NaCl, 5% [vol/vol] glycerol, 1 mM β-mercaptoethanol, 100 μg mL^−1^ RNase A, 1× protease inhibitors [Roche, complete mini, EDTA free], and 1 mg mL^−1^ lysozyme [Sigma-Aldrich]). Cell debris was removed by centrifugation at 35,000 × *g* for 45 min at 4°C. His-tagged Vn04 PB2-C protein was purified using nickel-nitrilotriacetic acid (Ni-NTA) agarose matrix (Qiagen) and eluted overnight at 4°C in elution buffer (50 mM Tris-HCl [pH 8.0], 200 mM NaCl, 5% [vol/vol] glycerol, 1 mM β-mercaptoethanol, 100 μg mL^−1^ RNase A, 1× protease inhibitors [Roche, complete mini, EDTA free], 1 mg mL^−1^ lysozyme [Sigma-Aldrich], 500 mM imidazole). The supernatants containing proteins were collected and concentrated using an Amicon Ultra centrifugal filter unit (Merck Millipore; NMWL of 30 kDa). The concentrated proteins were applied onto a Superdex 200 Increase 10/300 GL column (GE Healthcare) connected to an Äkta chromatography system (GE Healthcare) at 4°C equilibrated with a buffer consisting of 50 mM Tris-HCl (pH 8.0), 200 mM NaCl, 5% (vol/vol) glycerol, and 1 mM β-mercaptoethanol. The fractions containing proteins were pooled and concentrated as done previously. The molecular weight and the purity of the proteins were assessed by SDS-PAGE with Coomassie brilliant blue staining. The proteins were flash-frozen using liquid nitrogen and stored at −80°C.

### Viral infections.

Approximately 8 × 10^6^ HEK-293T cells were infected with influenza A/WSN/33 (H1N1) virus at a multiplicity of infection of 5 or were mock infected. After 12 h, cells were harvested by centrifugation at 180 × *g* for 5 min at 4°C and lysed at 4°C for 1 h in lysis buffer (50 mM HEPES-NaOH [pH 7.5], 200 mM NaCl, 0.5% [vol/vol] IGEPAL CA-630 [Sigma-Aldrich], 1 mM β-mercaptoethanol, 1× phenylmethylsulfonyl fluoride [PMSF], 1× protease inhibitor [Roche, complete mini, EDTA free]). Lysates were collected by centrifugation at 17,000 × *g* for 5 min at 4°C and used in a GST pulldown assay.

### GST pulldown assay.

Approximately 0.2 mg purified GST-tagged Rab11a or GST tag was incubated with preequilibrated glutathione Sepharose 4B beads (GE Healthcare) (100 μL slurry per 1 mL sample volume) at 4°C for 3 h in binding buffer (50 mM HEPES-NaOH [pH 7.5], 300 mM NaCl, 10% [vol/vol] glycerol, 8 mM MgCl_2_, 10 μM GTPγS [Abcam]). After binding, the glutathione Sepharose beads were washed in binding buffer. Resuspended beads were then incubated in binding buffer with either 0.06 mg purified heterotrimeric polymerase, 0.08 mg purified 627-NLS or PB2-C protein, or WSN-infected or mock-infected whole-cell lysates for 1 h at 4°C. After binding, the beads were washed five times in binding buffer and resuspended in the same buffer supplemented with 1 mM DTT and 0.1 mg 3C protease. After 1 h of incubation at 4°C, targeted protein complexes were cleared from the beads by centrifugation at 1,000 × *g* for 5 min at 4°C. GST pulldown protein fractions were analyzed by 12% SDS-PAGE with Coomassie blue staining and/or used for viral RNA and protein analyses.

### RNA extraction and primer extension analysis.

Total cellular RNA was extracted using TRI reagent (Sigma-Aldrich) according to manufacturer’s instructions. Viral RNA levels were analyzed using primer extension as previously described (46). In brief, RNA was reverse transcribed using ^32^P-labeled primers specific to positive- and negative-sense viral RNAs, with a primer specific to cellular 5S rRNA as a loading control. Transcripts were separated by 6% denaturing PAGE and visualized by phosphorimaging on an FLA-5000 scanner (Fuji). Analysis was carried out using ImageJ (47).

### Sample preparation for proteomics analysis.

Protein samples from GST pulldown assays were denatured in 4 M urea dissolved in 0.1 M ammonium bicarbonate at pH 8.5. Cysteines were reduced in 10 mM Tris(2-carboxyethyl)phosphine (TCEP; pH 7.0) for 30 min at room temperature and alkylated with 50 mM 2-chloroacetamide for 30 min at room temperature in the dark. Samples were then predigested using endoproteinase Lys-C at a ratio of 1 μg enzyme per 100 μg protein sample for 2 h shaking at 37°C. Urea was further diluted to 2 M in 0.1 M ammonium bicarbonate at pH 8.5, and CaCl_2_ was added at a final concentration of 2 mM. Finally, samples were digested with trypsin at a ratio of 1 μg enzyme per 40 μg protein sample overnight with shaking at 37°C. The trypsin reaction was quenched with 5% (vol/vol) formic acid, and samples were centrifuged for 30 min at 17,000 × *g* and 4°C to remove aggregates and undigested material. The digested peptides contained in the supernatant were desalted on handmade C_18_ stage tips before liquid chromatography-tandem mass spectrometry (LC-MS/MS) analysis.

### LC-MS/MS.

Peptides were separated by nanoliquid chromatography (Thermo Fisher Scientific; UltiMate RSLC 3000) coupled in line to a Q Exactive mass spectrometer equipped with an Easy-Spray source (Thermo Fisher Scientific). Peptides were trapped onto a C_18_ PepMac100 precolumn (300 μm [inside diameter] by 5 mm, 100 Å; Thermo Fisher Scientific) using solvent A (0.1% [vol/vol] formic acid, high-performance liquid chromatography [HPLC]-grade water). The peptides were further separated on an Easy-Spray RSLC C_18_ column (75-μm inside diameter, 50-cm length; Thermo Fisher Scientific) using a 60-min linear gradient of 15% to 35% solvent B (0.1% [vol/vol] formic acid in acetonitrile) at a flow rate of 200 nL min^−1^. The raw data were acquired on the mass spectrometer in a data-dependent acquisition (DDA) mode. Full-scan MS spectra were acquired in the Orbitrap (scan range, 350 to 1,500 *m/z*; resolution, 70,000; AGC target, 3e^6^; maximum injection time, 50 ms). The 10 most intense peaks were selected for higher-energy collision dissociation (HCD) fragmentation at 30% of normalized collision energy. HCD spectra were acquired in the Orbitrap at a resolution of 17,500, automatic gain control (AGC) target of 5e^4^, and maximum injection time of 60 ms, with fixed mass at 180 *m/z*. Charge exclusion was selected for unassigned and 1^+^ ions. The dynamic exclusion was set to 40 s.

### Database searching.

Tandem mass spectra were searched using SEQUEST HT within Proteome Discoverer PD1.4 (Thermo Fisher Scientific; version 1.4.0.288) against a database containing 20,418 protein entries combining protein sequences from Homo sapiens in which the endogenous Rab11a protein sequence had been replaced by GST-tagged Rab11a, IAV A/WSN/1933 (H1N1), and common contaminants. During database searches, cysteines (C) were considered to be fully carbamidomethylated (+57,0215, statically added), methionines (M) to be fully oxidized (+15,9949, dynamically added), and all N-terminal residues and lysines (K) to be acetylated (+42,0106, dynamically added). Two missed cleavages were permitted. Peptide mass tolerance was set at 50 ppm and 0.02 Da on the precursor and fragment ions, respectively. Protein identification was filtered at a false-discovery rate (FDR) below 1%.

### Quantitative proteomics and statistical analyses.

GST-Rab11a-interacting protein partners were identified by quantitative proteomic and statistical analyses, after sorting out proteins significantly enriched in the GST-Rab11a protein data set versus GST (negative control) under control and infected conditions. The quantitative analysis was based on a label-free quantitation method using normalized spectral abundance factor (NSAF), as a measure of relative protein abundance within the protein mixture. Spectral abundance factor (SAF) and NSAF values were calculated as previously described (48) as the number of spectral counts (PSM) that identify a protein divided by the protein length (L); the PSM/L value represents the SAF, which is then divided by the sum of PSM/L values for all proteins in the experiment. NSAF values were calculated after removal of contaminants such as keratins, endoproteinase Lys-C, trypsin, and immunoglobulins. For better visualization of the data, NSAF values were multiplied by 100. The statistical analysis was performed on NSAF values from two biological replicates, using a two-tailed *t* test.

### SEC-SAXS.

For size exclusion chromatography coupled with small-angle X-ray scattering (SEC-SAXS), Rab11a_1-173_ and Vn04 PB2-C were buffer exchanged to SEC-SAXS running buffer (50 mM Tris-HCl [pH 8.0], 150 mM NaCl, 1% [vol/vol] glycerol, 1 mM β-mercaptoethanol, 8 mM MgCl_2_, 10 μM GTPγS) and concentrated to 6 mg mL^−1^ and 10 mg mL^−1^, respectively. The SEC-SAXS experiments were performed at beamline B21, Diamond Light Source (Didcot, UK) (49). Samples were applied to a Shodex KW403-4F column (4.6-mm inside diameter by 300-mm length) which was connected into an Agilent 1200 HPLC system (Waters) at a flow rate of 0.16 mL min^−1^. The SEC-separated sample was exposed to X rays in a 1.6-mm diameter, 10-μm-thick quartz capillary flow cell, followed by data collection every 3 s with an EIGER 4M (Dectris) detector. The X-ray beam sizes were 1 mm (horizontal) by 0.5 mm (vertical) at the sample position and 0.08 mm (horizontal) by 0.07 mm (vertical) at the focal point (the detector surface). The wavelength of X ray was 0.95 Å, and the sample detector distance was 3.7 m. The measurement temperature was 20°C. Raw SAXS two-dimensional (2D) images were processed with the DAWN (https://dawnsci.org/) processing pipeline to produce normalized, integrated 1D unsubtracted SAXS curves. Analysis of SEC was carried out using Prism 9 (GraphPad). The background subtraction, averaging of the data, and all other subsequent SAXS analyses were performed in ScÅtter (http://www.bioisis.net).

### Data availability.

The mass spectrometry proteomics data have been deposited to the ProteomeXchange Consortium via the PRIDE (50) partner repository with the data set identifier PXD030058.

## References

[1] Taubenberger JK, Kash JC. 2010. Influenza virus evolution, host adaptation, and pandemic formation. Cell Host Microbe 7:440–451. 10.1016/j.chom.2010.05.009. 20542248 PMC2892379

[2] Eisfeld AJ, Neumann G, Kawaoka Y. 2015. At the centre: influenza A virus ribonucleoproteins. Nat Rev Microbiol 13:28–41. 10.1038/nrmicro3367. 25417656 PMC5619696

[3] Fodor E, Te Velthuis AJW. 2020. Structure and function of the influenza virus transcription and replication machinery. Cold Spring Harb Perspect Med 10:a038398. 10.1101/cshperspect.a038398. 31871230 PMC7334866

[4] Wandzik JM, Kouba T, Cusack S. 2021. Structure and function of influenza polymerase. Cold Spring Harb Perspect Med 11:a038372. 10.1101/cshperspect.a038372. 32341065 PMC8415296

[5] Te Velthuis AJW, Grimes JM, Fodor E. 2021. Structural insights into RNA polymerases of negative-sense RNA viruses. Nat Rev Microbiol 19:303–318. 10.1038/s41579-020-00501-8. 33495561 PMC7832423

[6] Lakdawala SS, Fodor E, Subbarao K. 2016. Moving on out: transport and packaging of influenza viral RNA into virions. Annu Rev Virol 3:411–427. 10.1146/annurev-virology-110615-042345. 27741407

[7] Fournier E, Moules V, Essere B, Paillart JC, Sirbat JD, Cavalier A, Rolland JP, Thomas D, Lina B, Isel C, Marquet R. 2012. Interaction network linking the human H3N2 influenza A virus genomic RNA segments. Vaccine 30:7359–7367. 10.1016/j.vaccine.2012.09.079. 23063835

[8] Noda T, Sagara H, Yen A, Takada A, Kida H, Cheng RH, Kawaoka Y. 2006. Architecture of ribonucleoprotein complexes in influenza A virus particles. Nature 439:490–492. 10.1038/nature04378. 16437116

[9] Gavazzi C, Yver M, Isel C, Smyth RP, Rosa-Calatrava M, Lina B, Moules V, Marquet R. 2013. A functional sequence-specific interaction between influenza A virus genomic RNA segments. Proc Natl Acad Sci USA 110:16604–16609. 10.1073/pnas.1314419110. 24067651 PMC3799358

[10] Dadonaite B, Gilbertson B, Knight ML, Trifkovic S, Rockman S, Laederach A, Brown LE, Fodor E, Bauer DLV. 2019. The structure of the influenza A virus genome. Nat Microbiol 4:1781–1789. 10.1038/s41564-019-0513-7. 31332385 PMC7191640

[11] Le Sage V, Kanarek JP, Snyder DJ, Cooper VS, Lakdawala SS, Lee N. 2020. Mapping of influenza virus RNA-RNA interactions reveals a flexible network. Cell Rep 31:107823. 10.1016/j.celrep.2020.107823. 32610124 PMC7372595

[12] Amorim MJ. 2018. A comprehensive review on the interaction between the host GTPase Rab11 and influenza A virus. Front Cell Dev Biol 6:176. 10.3389/fcell.2018.00176. 30687703 PMC6333742

[13] Pohl MO, Lanz C, Stertz S. 2016. Late stages of the influenza A virus replication cycle—a tight interplay between virus and host. J Gen Virol 97:2058–2072. 10.1099/jgv.0.000562. 27449792

[14] Welz T, Wellbourne-Wood J, Kerkhoff E. 2014. Orchestration of cell surface proteins by Rab11. Trends Cell Biol 24:407–415. 10.1016/j.tcb.2014.02.004. 24675420

[15] Horgan CP, McCaffrey MW. 2009. The dynamic Rab11-FIPs. Biochem Soc Trans 37:1032–1036. 10.1042/BST0371032. 19754446

[16] Chou Y-Y, Heaton NS, Gao Q, Palese P, Singer RH, Singer R, Lionnet T. 2013. Colocalization of different influenza viral RNA segments in the cytoplasm before viral budding as shown by single-molecule sensitivity FISH analysis. PLoS Pathog 9:e1003358. 10.1371/journal.ppat.1003358. 23671419 PMC3649991

[17] Lakdawala SS, Wu Y, Wawrzusin P, Kabat J, Broadbent AJ, Lamirande EW, Fodor E, Altan-Bonnet N, Shroff H, Subbarao K. 2014. Influenza A virus assembly intermediates fuse in the cytoplasm. PLoS Pathog 10:e1003971. 10.1371/journal.ppat.1003971. 24603687 PMC3946384

[18] Amorim MJ, Bruce EA, Read EK, Foeglein A, Mahen R, Stuart AD, Digard P. 2011. A Rab11- and microtubule-dependent mechanism for cytoplasmic transport of influenza A virus viral RNA. J Virol 85:4143–4156. 10.1128/JVI.02606-10. 21307188 PMC3126276

[19] Bruce EA, Digard P, Stuart AD. 2010. The Rab11 pathway is required for influenza A virus budding and filament formation. J Virol 84:5848–5859. 10.1128/JVI.00307-10. 20357086 PMC2876627

[20] Eisfeld AJ, Kawakami E, Watanabe T, Neumann G, Kawaoka Y. 2011. RAB11A is essential for transport of the influenza virus genome to the plasma membrane. J Virol 85:6117–6126. 10.1128/JVI.00378-11. 21525351 PMC3126513

[21] Momose F, Sekimoto T, Ohkura T, Jo S, Kawaguchi A, Nagata K, Morikawa Y. 2011. Apical transport of influenza A virus ribonucleoprotein requires Rab11-positive recycling endosome. PLoS One 6:e21123. 10.1371/journal.pone.0021123. 21731653 PMC3120830

[22] de Castro Martin IF, Fournier G, Sachse M, Pizarro-Cerda J, Risco C, Naffakh N. 2017. Influenza virus genome reaches the plasma membrane via a modified endoplasmic reticulum and Rab11-dependent vesicles. Nat Commun 8:1396. 10.1038/s41467-017-01557-6. 29123131 PMC5680169

[23] Ganti K, Han J, Manicassamy B, Lowen AC. 2021. Rab11a mediates cell-cell spread and reassortment of influenza A virus genomes via tunneling nanotubes. PLoS Pathog 17:e1009321. 10.1371/journal.ppat.1009321. 34473799 PMC8443049

[24] Alenquer M, Vale-Costa S, Etibor TA, Ferreira F, Sousa AL, Amorim MJ. 2019. Influenza A virus ribonucleoproteins form liquid organelles at endoplasmic reticulum exit sites. Nat Commun 10:1629. 10.1038/s41467-019-09549-4. 30967547 PMC6456594

[25] Han J, Ganti K, Sali VK, Twigg C, Zhang Y, Manivasagam S, Liang CY, Vogel OA, Huang I, Emmanuel SN, Plung J, Radoshevich L, Perez JT, Lowen AC, Manicassamy B. 2021. Host factor Rab11a is critical for efficient assembly of influenza A virus genomic segments. PLoS Pathog 17:e1009517. 10.1371/journal.ppat.1009517. 33970958 PMC8136845

[26] Avilov SV, Moisy D, Naffakh N, Cusack S. 2012. Influenza A virus progeny vRNP trafficking in live infected cells studied with the virus-encoded fluorescently tagged PB2 protein. Vaccine 30:7411–7417. 10.1016/j.vaccine.2012.09.077. 23063830

[27] Uhlig M, Passlack W, Eckel J. 2006. Identification and characterization of a novel variant in the highly conserved catalytic center of Rab11a. Eur J Med Genet 49:29–36. 10.1016/j.ejmg.2005.04.004. 16473307

[28] York A, Hengrung N, Vreede FT, Huiskonen JT, Fodor E. 2013. Isolation and characterization of the positive-sense replicative intermediate of a negative-strand RNA virus. Proc Natl Acad Sci USA 110:E4238–E4245. 10.1073/pnas.1315068110. 24145413 PMC3831450

[29] Fan H, Walker AP, Carrique L, Keown JR, Serna Martin I, Karia D, Sharps J, Hengrung N, Pardon E, Steyaert J, Grimes JM, Fodor E. 2019. Structures of influenza A virus RNA polymerase offer insight into viral genome replication. Nature 573:287–290. 10.1038/s41586-019-1530-7. 31485076 PMC6795553

[30] Nilsson BE, Te Velthuis AJW, Fodor E. 2017. Role of the PB2 627 domain in influenza A virus polymerase function. J Virol 91:e00523-17. 10.1128/JVI.00523-17. 28122973 PMC5355620

[31] Jagoe WN, Lindsay AJ, Read RJ, McCoy AJ, McCaffrey MW, Khan AR. 2006. Crystal structure of Rab11 in complex with Rab11 family interacting protein 2. Structure 14:1273–1283. 10.1016/j.str.2006.06.010. 16905101

[32] Bhagwat AR, Le Sage V, Nturibi E, Kulej K, Jones J, Guo M, Tae Kim E, Garcia BA, Weitzman MD, Shroff H, Lakdawala SS. 2020. Quantitative live cell imaging reveals influenza virus manipulation of Rab11A transport through reduced dynein association. Nat Commun 11:23. 10.1038/s41467-019-13838-3. 31911620 PMC6946661

[33] Shiba T, Koga H, Shin HW, Kawasaki M, Kato R, Nakayama K, Wakatsuki S. 2006. Structural basis for Rab11-dependent membrane recruitment of a family of Rab11-interacting protein 3 (FIP3)/Arfophilin-1. Proc Natl Acad Sci USA 103:15416–15421. 10.1073/pnas.0605357103. 17030804 PMC1622838

[34] Carrique L, Fan H, Walker AP, Keown JR, Sharps J, Staller E, Barclay WS, Fodor E, Grimes JM. 2020. Host ANP32A mediates the assembly of the influenza virus replicase. Nature 587:638–643. 10.1038/s41586-020-2927-z. 33208942 PMC7116770

[35] Vale-Costa S, Alenquer M, Sousa AL, Kellen B, Ramalho J, Tranfield EM, Amorim MJ. 2016. Influenza A virus ribonucleoproteins modulate host recycling by competing with Rab11 effectors. J Cell Sci 129:1697–1710. 10.1242/jcs.188409. 26940915

[36] Peng Q, Liu Y, Peng R, Wang M, Yang W, Song H, Chen Y, Liu S, Han M, Zhang X, Wang P, Yan J, Zhang B, Qi J, Deng T, Gao GF, Shi Y. 2019. Structural insight into RNA synthesis by influenza D polymerase. Nat Microbiol 4:1750–1759. 10.1038/s41564-019-0487-5. 31209309

[37] Reich S, Guilligay D, Pflug A, Malet H, Berger I, Crepin T, Hart D, Lunardi T, Nanao M, Ruigrok RW, Cusack S. 2014. Structural insight into cap-snatching and RNA synthesis by influenza polymerase. Nature 516:361–366. 10.1038/nature14009. 25409151

[38] Wandzik JM, Kouba T, Karuppasamy M, Pflug A, Drncova P, Provaznik J, Azevedo N, Cusack S. 2020. A structure-based model for the complete transcription cycle of influenza polymerase. Cell 181:877–893.e21. 10.1016/j.cell.2020.03.061. 32304664

[39] Paterson D, Fodor E. 2012. Emerging roles for the influenza A virus nuclear export protein (NEP). PLoS Pathog 8:e1003019. 10.1371/journal.ppat.1003019. 23236273 PMC3516560

[40] Robb NC, Chase G, Bier K, Vreede FT, Shaw PC, Naffakh N, Schwemmle M, Fodor E. 2011. The influenza A virus NS1 protein interacts with the nucleoprotein of viral ribonucleoprotein complexes. J Virol 85:5228–5231. 10.1128/JVI.02562-10. 21411538 PMC3126214

[41] Sha TW, Weber M, Kasumba DM, Noda T, Nakano M, Kato H, Fujita T. 2020. Influenza A virus NS1 optimises virus infectivity by enhancing genome packaging in a dsRNA-binding dependent manner. Virol J 17:107. 10.1186/s12985-020-01357-3. 32677963 PMC7367362

[42] Hutchinson EC, Charles PD, Hester SS, Thomas B, Trudgian D, Martinez-Alonso M, Fodor E. 2014. Conserved and host-specific features of influenza virion architecture. Nat Commun 5:4816. 10.1038/ncomms5816. 25226414 PMC4167602

[43] Hoffmann E, Neumann G, Kawaoka Y, Hobom G, Webster RG. 2000. A DNA transfection system for generation of influenza A virus from eight plasmids. Proc Natl Acad Sci USA 97:6108–6113. 10.1073/pnas.100133697. 10801978 PMC18566

[44] Bieniossek C, Imasaki T, Takagi Y, Berger I. 2012. MultiBac: expanding the research toolbox for multiprotein complexes. Trends Biochem Sci 37:49–57. 10.1016/j.tibs.2011.10.005. 22154230 PMC7127121

[45] Hengrung N, El Omari K, Serna Martin I, Vreede FT, Cusack S, Rambo RP, Vonrhein C, Bricogne G, Stuart DI, Grimes JM, Fodor E. 2015. Crystal structure of the RNA-dependent RNA polymerase from influenza C virus. Nature 527:114–117. 10.1038/nature15525. 26503046 PMC4783868

[46] Robb NC, Smith M, Vreede FT, Fodor E. 2009. NS2/NEP protein regulates transcription and replication of the influenza virus RNA genome. J Gen Virol 90:1398–1407. 10.1099/vir.0.009639-0. 19264657

[47] Schneider CA, Rasband WS, Eliceiri KW. 2012. NIH Image to ImageJ: 25 years of image analysis. Nat Methods 9:671–675. 10.1038/nmeth.2089. 22930834 PMC5554542

[48] Florens L, Carozza MJ, Swanson SK, Fournier M, Coleman MK, Workman JL, Washburn MP. 2006. Analyzing chromatin remodeling complexes using shotgun proteomics and normalized spectral abundance factors. Methods 40:303–311. 10.1016/j.ymeth.2006.07.028. 17101441 PMC1815300

[49] Cowieson NP, Edwards-Gayle CJC, Inoue K, Khunti NS, Doutch J, Williams E, Daniels S, Preece G, Krumpa NA, Sutter JP, Tully MD, Terrill NJ, Rambo RP. 2020. Beamline B21: high-throughput small-angle X-ray scattering at Diamond Light Source. J Synchrotron Radiat 27:1438–1446. 10.1107/S1600577520009960. 32876621 PMC7467336

[50] Perez-Riverol Y, Csordas A, Bai J, Bernal-Llinares M, Hewapathirana S, Kundu DJ, Inuganti A, Griss J, Mayer G, Eisenacher M, Pérez E, Uszkoreit J, Pfeuffer J, Sachsenberg T, Yilmaz S, Tiwary S, Cox J, Audain E, Walzer M, Jarnuczak AF, Ternent T, Brazma A, Vizcaíno JA. 2019. The PRIDE database and related tools and resources in 2019: improving support for quantification data. Nucleic Acids Res 47:D442–D450. 10.1093/nar/gky1106. 30395289 PMC6323896

